# Clean Your Hands 5th May 2017: 'Fight antibiotic resistance - it's in your hands'

**DOI:** 10.1186/s13756-017-0196-x

**Published:** 2017-05-05

**Authors:** Ermira Tartari, Daniela Pires, Didier Pittet

**Affiliations:** 1 Infection Control Programme and WHO Collaborating Centre on Patient Safety - Infection Control & Improving Practices, University of Geneva Hospitals and Faculty of Medicine, 4 Rue Gabrielle-Perret-Gentil, 1211 Geneva, Switzerland; 2 Infection Control Unit, Mater Dei Hospital, Msida, Malta; 3 Department of Infectious Diseases, Centro Hospitalar Lisboa Norte and Faculdade de Medicina da Universidade de Lisboa, Lisbon, Portugal

Preventing healthcare-associated infections and reducing their avoidable impact on health systems is critical today to make facilities safer for patients worldwide [1]. In addition, the increasing public health burden of antimicrobial resistance (AMR) urges to action [2]. Stronger political commitment in reducing AMR was highlighted at the last United Nations General Assembly in September 2016 in New York.

Hand hygiene is at the center of effective infection prevention and control (IPC) to combat AMR spread [3]. The World Health Organization (WHO) recently issued guidelines on the Core Components of effective IPC programmes [1]. Their implementation will allow for strong, resilient health systems in all settings. The guidelines include the application of a multimodal strategy that consists in achieving system change (infrastructure and resources), raising awareness, education and training, monitoring and timely feedback and a patient safety culture that includes visibly committed leadership. This approach improves hand hygiene, reduces infections and saves lives [4]. Therefore, on the occasion of the upcoming 5^th^ May 2017 Global Annual Hand Hygiene Day, WHO urges policy makers, top-level managers, IPC specialists and other health professionals to focus on the fight against AMR spread, by building ever stronger hand hygiene and IPC programmes (Table 1).

**Table 1:** 5^th^ May 2017 key World Health Organization campaign messages

**Health workers:** “Clean your hands at the right times and stop the spread of antibiotic resistance.”
**Hospital Chief Executive Officers and Administrators:** “Lead a year-round infection prevention and control programme to protect your patients from resistant infections."
**Policy-makers:** "Stop antibiotic resistance spread by making infection prevention and hand hygiene a national policy priority."
**IPC leaders:** "Implement WHO’s Core Components for infection prevention, including hand hygiene, to combat antibiotic resistance.”

We encourage health facilities worldwide to endorse the WHO's 5^th^ May 2017 campaign [5] and further improve hand hygiene, fight antibiotic resistance and commit to progressing towards adherence with all core components of IPC programmes.

Let’s fight antibiotic resistance together; it's in our hands.


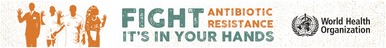


## Abbreviations

AMR: Antimicrobial resistance
IPC: Infection prevention and control
WHO: World Health Organization.
